# Hepatoprotective Mechanisms Induced by Spinach Methanolic Extract in Rats with Hyperglycemia—An Immunohistochemical Analysis

**DOI:** 10.3390/antiox12112013

**Published:** 2023-11-17

**Authors:** Javier Flores-Estrada, Agustina Cano-Martínez, Álvaro Vargas-González, Vicente Castrejón-Téllez, Jorge Cornejo-Garrido, Martín Martínez-Rosas, Verónica Guarner-Lans, María Esther Rubio-Ruíz

**Affiliations:** 1 División de Investigación, Hospital Juárez de México, Mexico City 07760, Mexico; 2 Departamento de Fisiología, Instituto Nacional de Cardiología Ignacio Chávez, Mexico City 14080, Mexico; agustina.cano@cardiologia.org.mx (A.C.-M.); alvaro.vargas@cardiologia.org.mx (Á.V.-G.); vicente.castrejon@cardiologia.org.mx (V.C.-T.); martin.martinez@cardiologia.org.mx (M.M.-R.); veronica.guarner@cardiologia.org.mx (V.G.-L.); esther.rubio@cardiologia.org.mx (M.E.R.-R.); 3 Laboratorio de Biología Celular y Productos Naturales, Escuela Nacional de Medicina y Homeopatía (ENMH), Instituto Politécnico Nacional, Mexico City 07320, Mexico; jcornejog@ipn.mx

**Keywords:** spinach methanolic extract (SME), liver damage, oxidative stress, inflammation, fibrosis, parenchymal (PQ) cells, non-parenchymal (nPQ) cells, immunohistochemistry

## Abstract

Spinach methanolic extract (SME) has a hepatoprotective effect due to its polyphenolic antioxidants; however, its action in parenchymal (PQ) and non-parenchymal (nPQ) cells remains unknown. This study investigates the hepatoprotective effect of SME on streptozotocin-induced hyperglycemic rats (STZ), focusing on immunohistochemical analyses. **Methods**: The extract was prepared, and the total polyphenols and antioxidant activity were quantified. Adult male Wistar rats were divided into four groups (*n* = 8): normoglycemic rats (NG), STZ-induced hyperglycemic (STZ), STZ treated with 400 mg/kg SME (STZ-SME), and NG treated with SME (SME) for 12 weeks. Serum liver transaminases and lipid peroxidation levels in tissue were determined. The distribution pattern and relative levels of markers related to oxidative stress [reactive oxygen species (ROS), superoxide dismutase-1, catalase, and glutathione peroxidase-1], of cytoprotective molecules [nuclear NRF2 and heme oxygenase-1 (HO-1)], of inflammatory mediators [nuclear NF-κB, TNF-α], proliferation (PCNA), and of fibrogenesis markers [TGF-β, Smad2/3, MMP-9, and TIMP1] were evaluated. **Results**: SME had antioxidant capacity, and it lowered serum transaminase levels in STZ-SME compared to STZ. It reduced NOX4 staining, and lipid peroxidation levels were related to low formation of ROS. In STZ-SME, the immunostaining for antioxidant enzymes increased in nPQ cells compared to STZ. However, enzymes were also localized in extra and intracellular vesicles in STZ. Nuclear NRF2 staining and HO-1 expression in PQ and nPQ were higher in STZ-SME than in STZ. Inflammatory factors were decreased in STZ-SME and were related to the percentage decrease in NF-κB nuclear staining in nPQ cells. Similarly, TGF-β (in the sinusoids) and MMP-9 (in nPQ) were increased in the STZ-SME group compared to the other groups; however, staining for CTGF, TIMP1, and Smad2/3 was lower. **Conclusions**: SME treatment in hyperglycemic rats induced by STZ may have hepatoprotective properties due to its scavenger capacity and the regulation of differential expression of antioxidant enzymes between the PQ and nPQ cells, reducing inflammatory and fibrogenic biomarkers in liver tissue.

## 1. Introduction

Diabetes mellitus (DM) is a metabolic disorder that leads to chronic hyperglycemia. This condition arises due to the body’s inability to produce insulin (DM type 1) or to respond to this hormone (DM type 2) [1]. Chronic hyperglycemia in DM can significantly impact the liver. It may lead to non-alcoholic fatty liver disease (NAFLD), where fat accumulates in the liver and can progress to more severe forms like non-alcoholic steatohepatitis (NASH), cirrhosis, and eventually, hepatocellular carcinoma (HCC) [2,3,4]. Fatty liver disease is becoming the leading determinant of liver transplantation, liver-related morbidity, and mortality when left without external intervention [5]. Further studies are required to understand the harmful potential of liver disease and to fully understand the underlying mechanisms. Furthermore, no approved targeted treatments are currently available, and the high incidence and impact on human health are concerning [6].

Recent studies suggest that diabetic hypertriglyceridemia is associated with oxidative stress, inflammation, and endoplasmic reticulum (ER) stress [7,8]. Hyperglycemia can activate NADPH oxidase 4 (NOX4) and produce reactive oxygen species (ROS) in the liver. Augmented ROS result in the death of hepatic parenchymal (PQ) cells and in the release of pro-inflammatory factors from non-parenchymal (nPQ) cells, such as hepatic stellate cells (HSCs) and Kupffer cells (KCs) [9,10,11,12]. On the other hand, the ROS levels can be regulated by activating the endogenous antioxidant enzymatic systems, including superoxide dismutase (SOD), catalase (CAT), glutathione peroxidase (GPx), glutathione reductase (GSR), and glutathione-S-transferase (GST) [13]. Interestingly, ROS trigger nuclear translocation of NRF2 with expression of HO-1, reducing oxidative stress, inflammation, and fibrosis [14].

Pro-inflammatory factors such as tumoral necrosis-alpha factor (TNF-α), interleukin-1β (IL-1β), interleukin-6 (IL-6), and the transformant growth factor β1 (TGF-β1) contribute to liver fibrosis induced by oxidative stress. nPQ cells such as HSCs release TGF-β1 and stimulate the production of myofibroblasts by the TGF-β1/Smad pathway by promoting extracellular matrix synthesis and the secretion of antiproteases, such as tissue inhibitors of metalloproteinases (TIMP) [15,16]. TIMP1 inhibits the breakdown of the extracellular matrix, usually induced by metalloproteinase 9 (MMP-9) [17]. Therefore, liver fibrosis, similarly to oxidative stress, is a complex process involving various mechanisms, inflammation and tissue remodeling. Consequently, developing effective treatment strategies against liver fibrosis can be challenging [6,18,19].

Natural sources of antioxidant polyphenols, such as spinach leaves (*Spinacia oleracea* L.), can help prevent liver damage caused by oxidative stress. Spinach leaves have a high nutritional value as they contain minerals, vitamins, polyphenols (carotenoids and flavonoids), and other phytochemicals that have hepatoprotective effects in animal models. These compounds reduce oxidative stress and inflammatory factors and improve glucose levels, some histological features of NAFLD, and specific liver damage markers [20,21,22,23,24,25,26]. On the other hand, the administration of a spinach leaf extract attenuated cardiac damage associated with reduced levels of TNF-α, IL-1β, and IL-6 in models of myocardial necrosis and animals on a high-fat, high-fructose diet [27,28]. In this scenario, consuming the polyphenolic antioxidants of spinach could be an alternative therapeutic strategy to address liver damage induced by chronic hyperglycemia. However, more research is needed to fully understand its benefits in humans.

An animal model study was used to investigate the effect of methanolic extract from spinach leaves on liver damage caused by hyperglycemia induced by streptozotocin. Our primary objective was to assess the antioxidant properties of the spinach extract and the distribution of markers related to oxidative, inflammatory, and fibrotic factors in hepatocytes (parenchymal cells; PQ) and nPQ cells.

## 2. Materials and Methods

### 2.1. Total Polyphenols and Antioxidant Activity of the Methanolic Extract of Spinach

Methanolic extract of spinach leaves (*Spinacia oleracea* L.) (SME) was obtained according to the procedure previously described [29]; fresh leaves were harvested in the winter season in Puebla, Mexico. The SME was then stored in the dark at 4 °C.

To determine the phenolic contents in the SME, we followed the procedure outlined by Prior et al. [30] with slight modifications, using a Folin–Ciocalteu phenolic reagent. Twelve microliters of varying SME concentrations (100, 50, 25, 12.5, and 6.25 mg/mL) were mixed with 270 µL of Folin–Ciocalteu reagent (1:4) and incubated for 5 min at room temperature (RT). Then, 120 μL of sodium carbonate (15%) was added, and the mixture was allowed to react for 45 min at RT. The absorbance was subsequently measured at 752 nm against the blank solution. The standard curve was established with quercetin preparations (1.0, 0.5, 0.25, 0.125, and 0.0625 mg/mL), and the data were converted to mg of quercetin per g of SME. The results were calculated from the average of three repetitions.

To assess the antioxidant activity of varying concentrations of SME (ranging from 0–100 mg/mL), we utilized the well-established DPPH (2,2-diphenyl-1-picryl-hydrazyl) method, as described by Brand-Williams et al. [31]. The process involved incubating the solution for 30 min at RT, followed by measurement of DPPH reduction to DPPH-H (diphenyl-picrylhydrazine) through absorbance readings at 515 nm. The antioxidant activity was then determined by measuring the equivalent mg of Trolox per 100 g of SME, which was calculated using a Trolox calibration curve ranging from 0 to 100 mg/L. The antioxidant activity of SME was also determined according to the method described by Re et al. [32] via the cationic bleaching assay of the ABTS radical (2,2′azinobis-(3-ethylbenzothiazoline)-6-sulfonic acid).

### 2.2. Animal Model and Experimental Design

Male Wistar rats weighing 280 ± 10 g were fasted for 8 h. The induction of hyperglycemia was performed via the intraperitoneal administration of a dose of streptozotocin (60 mg/kg body weight) prepared in sodium citrate buffer (10 mM, pH 4.5) (STZ; Sigma-Aldrich, Inc. St. Louis, MO, USA) [33]. Three days after administration, capillary blood glucose levels were measured (One touch ultra mini; Roche diagnostic, GmbH, Mannheim, Germany) in blood taken from the rats’ tails. Rats with blood glucose levels above 350 mg/dL were recruited. 

The rats were divided as follows (*n* = 8): normoglycemic (NG) rats treated with vehicle (drinking water by gastric gavage), STZ-induced hyperglycemic rats (STZ), STZ-treated with SME at 400 mg/kg (STZ-SME), and SME-treated NG (SME). The glycemic levels of each group were monitored every seven days. LabDiet 5001^®^ (PMI Nutrition INT’L., LLC, Shoreview, MN, USA) and tap water were provided ad libitum. Animals were given assigned doses daily for 12 weeks. Finally, rats were pre-anesthetized with ketamine–xylazine before blood sampling and sacrificed by administering an overdose of pentobarbital through a cannula inserted into the vena cava. The livers were promptly perfused with heparinized phosphate buffer (10 IU/mL; 100 mM, pH 7.4) and then dissected.

The 400 mg/kg dose of SME was chosen because 7 g of SME can be extracted from 100 g of fresh spinach, equivalent to the amount consumed daily by an average person weighing 70 kg in the American diet, translating to 100 mg of extract/kg of body weight [34]. However, it is recommended to increase the consumption of natural extracts up to 6.4 times for comparison studies with humans due to differences in rat metabolism [35]. In addition, the anti-inflammatory effects of SME were reported in a myocardial necrosis model in Wistar rats at a similar dose [28]. 

The animals received human care following the guidelines approved by the Research Ethics Committee of the National Polytechnic Institute (CONBIOETICA/09/CEI/002/20190327) and the Research Committee of the Hospital Juárez de México (HJM0713/19-1).

### 2.3. Serum Biochemical Parameters and Transaminases Activities

Glucose, total cholesterol, and triglyceride concentrations were measured using commercial enzymatic assays (RANDOX Laboratories, County Antrim, UK). Insulin was determined using a commercial radioimmunoassay (RIA) Kit specific for rats (Linco Research, Inc.; EMD Millipore, St. Louis, MI, USA). Glutamic-oxaloacetic transaminase (AST), glutamic pyruvic transaminase (ALT), and alkaline phosphatase (ALP) activities were determined using a kit from Pointe Scientific Inc. (Canton, MI, USA). The biochemical parameters were adjusted using the values of the NG group as a reference point for normalization.

### 2.4. Lipid Peroxidation Assay

The liver tissue underwent the lipid peroxidation assay using thiobarbituric acid reactive substances (TBARSs) and following the manufacturer’s instructions (OXItek-TBARS assay kit, Enzo Life Sciences, Farmingdale, NY, USA). Ten mg of tissue (*n* = 8) were homogenized in liquid nitrogen, and 1.0 mL of PBS 1× was added. After determining the total protein concentration in the tissue homogenate via the Bradford method, the concentration adjustment to 1.0 mg/mL was made. The reaction mixture was incubated at 95 °C for one hour and chilled on ice for 10 min. Then, it was centrifuged at 3000 rpm at 4 °C for 15 min, and the malondialdehyde (MDA) (nmol/mg protein) concentration was measured in the supernatant by absorbance at 532 nm (EON, BioTek Instruments, Inc., Winooski, VT, USA). The MDA concentration was calculated based on the mean MDA absorbance of a standard curve.

### 2.5. Detection of ROS by CellROX^®^

Fresh liver tissues were embedded in Tissue-Tek^®^ O.C.T. Compound (Sakura Finetek USA, Inc., Torrance, CA, USA). The tissues were then frozen and cut into 10 µm thick sections using a cryostat (Microtome Plus TM cryostat TBS, Urbana, IL, USA). The sections were mounted on electrocharged slides and washed with 1× PBS. Next, they were incubated with CellROX^®^ Deep Red Reagent at a concentration of 50 μM/mL (Molecular Probes by Life Technologies in Carlsbad, Carlsbad, CA, USA). This incubation occurred in a humid chamber at 37 °C for 30 min in darkness. After five washes with PBS, the nuclei were counterstained with 4′,6-diamidino-2-fenilindol (DAPI). The visualization and image acquisition were conducted on a FLoid^®^ Cell Imaging Station (Life Technologies Corporation, Carlsbad, CA, USA). For staining intensity measurements, 16 images were captured from four animals in each group. The Image Pro-Premier 9 software by Media Cybernetics was used.

### 2.6. Evaluation of Tissue Structure and Immunohistochemistry Markers

The liver tissues were first preserved in neutral formalin, dehydrated with graded alcohols, and finally embedded in paraffin. Histological sections of 2 μm thickness were placed on electrocharged slides, dewaxed, and rehydrated using a citrate buffer solution to recover the antigen (K035; Diagnostic BioSystems, Pleasanton, CA, USA). After that, the sections were blocked with Background Blocker (K023, Diagnostic BioSystems, Pleasanton, CA, USA) for 60 min at RT in a humid chamber (Shandon coverplate^®^; Shandon Sequenza; Thermo Scientific, Kalamazoo, MI, USA). The study used primary antibodies including NADPH-NOX4 (ab133303; Abcam PLC, Cambridge, UK), catalase (CAT; sc -271803, Santa Cruz Biotechnology, Inc., Dallas, TX, USA), superoxide dismutase 1 (SOD1; sc-271014, Santa Cruz Biotechnology Inc., Dallas, TX, USA), glutathione peroxidase (GPx1, sc-133160; Santa Cruz Biotechnology Inc., Dallas, TX, USA), nuclear factor erythroid 2-related factor (NFR2, sc-722; Santa Cruz Biotechnology Inc., Dallas, TX, USA); heme oxygenase 1 (HO-1; sc-7695, Santa Cruz Biotechnology Inc., Dallas, TX, USA), nuclear factor κ-light-chain-enhancer of activated B cells p65 subunit (NF-κBp65; sc-8008; Santa Cruz Biotechnology Inc., Dallas, TX, USA), tumor necrosis factor-α (TNF-α; ab1793, Abcam PLC Cambridge, UK), proliferating cell nuclear antigen (PCNA; 13-3900, Invitrogen Biotechnology, Waltham, MA, USA), transforming growth factor β1 (TGF-β1; sc-31609, Santa Cruz Biotechnology Inc., Dallas, TX, USA), connective tissue growth factor (CTGF; sc-365970, Santa Cruz Biotechnology Inc., Dallas, TX, USA), Smad proteins (mothers against decantaplegic homolog 2/3) (SMAD2/3; ab63399, Abcam PLC Cambridge, UK), metalloproteinase 9 (MMP-9; sc-393859, Santa Cruz Biotechnology Inc., Dallas, TX, USA); tissue inhibitor of metalloproteinase 1 (TIMP1; sc-516102, Santa Cruz Biotechnology Inc., Dallas, TX, USA). Primary antibodies were diluted 1:50 to 1:200 and incubated overnight at 4 °C. Subsequently, the sections were incubated for 2 h at RT with the corresponding horseradish peroxidase (HRP)-conjugated secondary antibodies (1:100; goat anti-rabbit; goat anti-mouse; Abcam PLC, Cambridge, UK). Afterward, the sections were stained with the chromogen DAB (3,3′-Diaminobenzidine, K047, Diagnostic BioSystems) and then counterstained with hematoxylin. The histological image captures were performed with a Carl Zeiss microscope (Axio Imager.A2, Carl Zeiss Microscopy GmbH, Jena, Germany) equipped with a built-in camera (Axiocam ICc5; Carl Zeiss Microscopy GmbH, Oberkochen, Germany).

Although no specific markers for PQ and nPQ cells were used, the criterion for distinguishing between the two types of cells was based on the cell’s location, size, and shape. PQ corresponds to larger hepatocytes with a “hexagonal” shape. In contrast, nPQ cells [liver sinusoidal endothelial cells (LSECs), hepatic stellate cells (HSCs), cholangiocytes and Kupffer cells, macrophages, dendritic cells, and hepatic sinusoidal cells (LSECs)] are smaller in size, with a non-geometric shape and located in the sinusoidal lumen and space of Disse [36]. Therefore, we classified non-parenchymal cell staining as everything that is not a hepatocyte, and this includes any cell that may be present in portal areas and sinusoids. 

### 2.7. Statistical Analysis

The intensity of staining was evaluated using the integrated optical density (IOD) (lum/pixel2), which was calculated by multiplying the sum of total area per mean density using Image-Pro Plus software version 6.0 (Media Cybernetics, Inc., Rockville, MD, USA). 

The percentage of positive cells was calculated as the ratio of stained cells (nuclei or cytoplasm) to the total cells per field. For SOD1, NRF2, and NF-kB, nuclear staining in PQ and nPQ cells was quantified separately, along with the quantification of cytoplasmic SOD1 staining in PQ cells. The data were captured independently by two observers. The fields were randomly selected under a microscope with 200× magnification. At least 80 fields per group of animals (*n* = 8; 10 fields per animal) were used. The Kolmogorov–Smirnov test was used to determine the data distribution, resulting in a non-parametric distribution. The statistical analysis was performed using GraphPad Prism software (La Jolla, CA, USA; version 8.0). A one-way ANOVA test followed by the Kruskal–Wallis test was performed in all cases. The results were considered statistically significant when *p* < 0.05.

## 3. Results

### 3.1. Antioxidant Activity of the Methanolic Extract of Spinach

**Figure 1:**
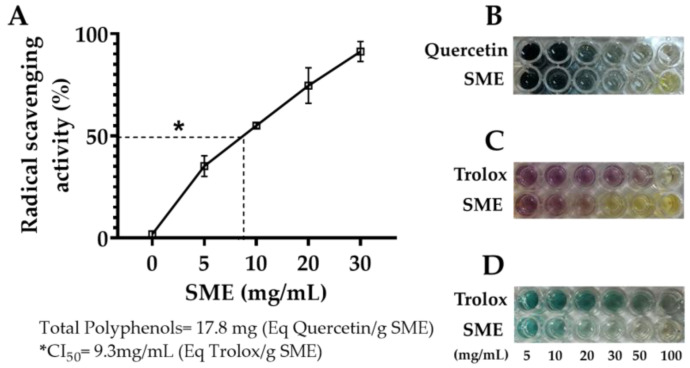
Scavenger activity and total polyphenols measurements of spinach methanolic extract (SME). Panel (**A**) illustrates the scavenging activity percentage concerning the concentration of SME (mg/mL). Panel (**B**) represents the Folin–Ciocalteu, 2,2-diphenyl-1-picryl-hydrazyl (DPPH) assay, which is used to measure the total content of polyphenols (equivalent to mg Quercetin/g of SME). Panel (**C**,**D**) show two 2,2′azinobis-(3-ethylbenzothiazoline)-6-sulfonic acid (ABTS) assays to determine antioxidant activity (Eq Trolox/g of SME). * IC50 denotes 50% free radical inhibitory activity.

The level of polyphenols and the antioxidant activity of spinach extract may vary. Therefore, the total amount of polyphenols in the SME and their scavenging activity were measured. As shown in Figure 1A, the total polyphenols content in SME was 17.8 ± 0.05 mg/Eq g quercetin, while its free radical 50% inhibitory concentration (IC50) was 9.3 ± 0.5 mg/mL. The antioxidant activity percentage increased with the increase in the concentration of SME, and 90% of its activity was achieved at 30 mg/mL of SME.

### 3.2. Effect of SME on Serum Biochemical Parameters and Enzyme Levels

As expected, the treatment with STZ significantly decreased the insulin concentration, which is not corrected with the administration of SME. Table 1 shows that the animals treated with STZ had significantly increased blood glucose levels (*p* < 0.05); the glucose concentrations remained constant after the treatment with SME. At the same time, the triglyceride levels are high in the STZ group compared to NG animals, and the SME administration reduced the triglyceride levels by 50%. No significant difference in total cholesterol concentrations between the experimental groups was observed. In the NG group, SME administration did not affect the serum parameters. 

Transaminases AST and ALT and phosphatase ALP levels were analyzed as they can indicate liver damage when found to be elevated. The values are shown in Table 1. The serum enzyme levels were significantly increased in the STZ group compared to NG and SME groups (*p* < 0.05); however, AST, ALT, and ALP levels were significantly lower in STZ-SME compared to STZ rats. The enzyme levels in STZ-SME did not reach the basal levels of the NG and SME groups, but only ALT levels were significantly higher than in the NG and SME groups (*p* < 0.05).

### 3.3. Reduction of Oxidative Stress Markers by SME in Liver Tissue

**Figure 2:**
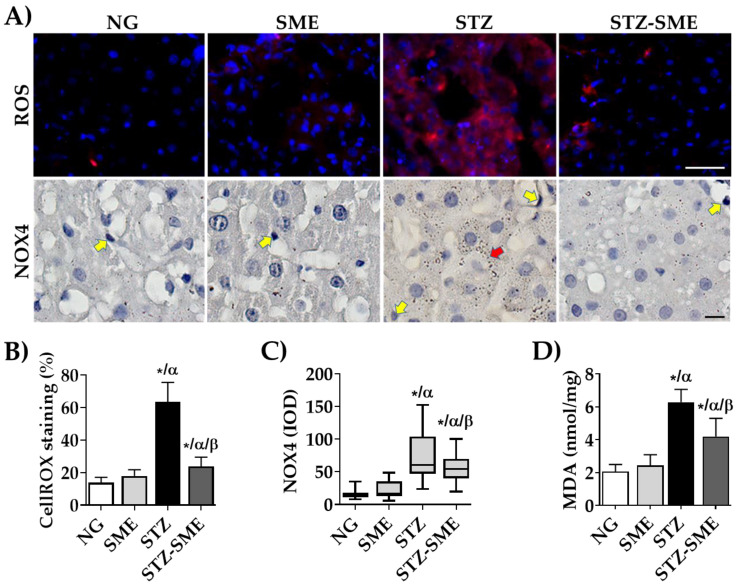
Effect of SME on oxidative stress. Panel (**A**) shows the distribution of reactive oxygen species (ROS) by immunofluorescence and Nicotinamide adenine dinucleotide phosphate oxidase 4 (NOX4) staining in livers of normoglycemic (NG) rats, NG-treated with SME at 400 mg/kg (SME), in rats with streptozotocin (STZ)-induced hyperglycemia, and STZ-treated with SME (STZ-SME). Panel (**B**) shows the percentage of CellROX staining (mean ± SD; *n* = 4). Panel (**C**) shows the integrated optical density (IOD, lum/pixel^2^) of NOX4 in a box-and-whisker plot (median, first-third quartile, minimum-maximum value; *n* = 8). In panel (**D**), the lipid peroxidation levels were measured by malondialdehyde (MDA) formation (mean ± SD; *n* = 8). The yellow and red arrows indicate positive stains in parenchymal cells (PQ) and non-parenchymal (nPQ) cells. * *p* < 0.01 compared to NG; α *p* < 0.05 compared to SME; and β *p* < 0.05 compared to STZ. Scale bar = 50 μm.

To study the impact of the antioxidant activity of SME on rat liver tissue damaged by oxidative stress, the formation of ROS (by CellROX^®^ staining) and the distribution of NOX4 intensity staining were evaluated. As shown in Figure 2A,B, STZ-SME had lower ROS levels than STZ (*p* < 0.01). Additionally, the ROS intensities in the NG and SME groups were lower than STZ-SME (*p* < 0.01).

Figure 2A shows that NOX4 immunostaining was found in both hepatocytes (parenchymal cells; PQ) and non-parenchymal (nPQ) cells. In all groups, NOX4 was present in the cytoplasm of hepatocytes. Moreover, staining was also found in the membranous and perinuclear spaces in STZ and STZ-SME. Notably, the staining intensity in STZ-SME was lower than STZ group (*p* < 0.01). Furthermore, the staining intensities in NG and SME were lower than in STZ and STZ-SME (*p* < 0.01) (Figure 2C).

The observed decrease in ROS and the low protein expression of NOX4 in liver tissue from the STZ-SME group were significantly associated with elevated lipid peroxidation, as determined by examining the levels of MDA formation (Figure 2D). The STZ group exhibited a MDA significant increase (*p* < 0.05) in comparison to the other groups, while STZ-SME levels were higher than those observed in the NG and SME groups (*p* < 0.05). No significant differences were observed between NG and SME.

### 3.4. The Impact of SME on the Distribution of the Expression of Antioxidant Enzymes

**Figure 3:**
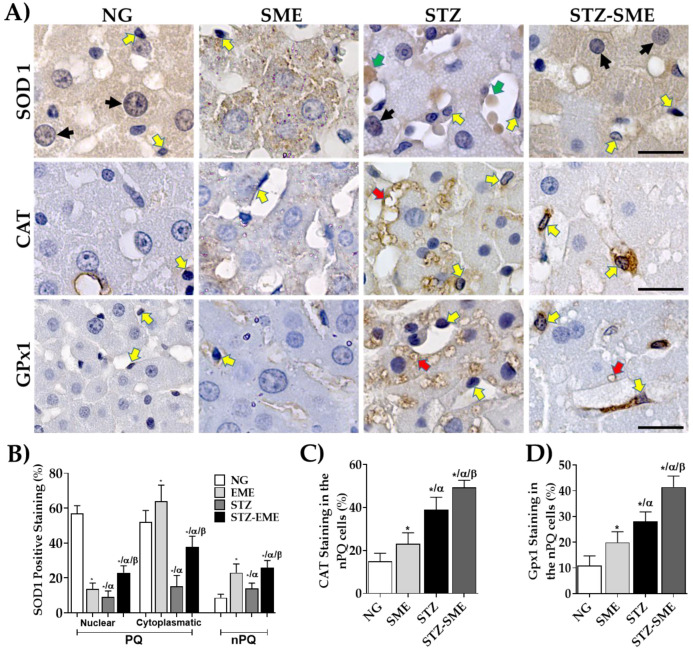
Effect of SME in the distribution of endogenous antioxidant enzymes in liver tissue. Panel (**A**) shows representative micrographs of immunostaining for superoxide dismutase 1 (SOD1), catalase (CAT), and glutathione peroxidase (Gpx1). The staining percentage for each immunostaining in the NG, SME, STZ, and STZ-SME groups is presented as mean ± SD in panels (**B**–**D**) (*n* = 8). The yellow arrows show positive staining in nPQ cells. Black arrows denote nuclear positive staining in PQ cells. The green and red arrows indicate potential extracellular vesicles and intracellular vacuoles, respectively. * *p* < 0.05 compared to NG; α *p* < 0.05 compared to SME; and β *p* < 0.05 compared to STZ. Scale bar = 20 μm.

Oxidative stress results from the augmented ROS and its counteraction by the endogenous antioxidant enzyme levels. To determine whether SME has effects on the expression of superoxide dismutase 1 (SOD1), catalase (CAT), and glutathione peroxidase 1 (Gpx1), their localization and distribution were evaluated in liver PQ and nPQ cells. The distribution and localization of antioxidant enzymes differed in the PQ and nPQ cells of the groups studied (Figure 3A). Regarding SOD1 staining, the NG group showed more intense nuclear staining in PQ cells (*p* < 0.05), while the SME group showed mainly cytoplasmic staining (*p* < 0.05). In nPQ cells, SOD1 was increased in SME compared to NG (*p* < 0.05). In hyperglycemic rats, the treatment with SME compared to STZ, SOD1 staining was distributed in both nPQ and cytoplasm of PQ cells, with a significant increase (*p* < 0.05). However, the STZ-SME group showed lower staining in PQ cells than in NG and SME. On the other hand, in nPQ cells, the percentage of SOD1 staining was higher (*p* < 0.05) than in the other groups (Figure 3A,B). Additionally, in STZ, SOD1 was primarily found in vacuoles outside of cells located in the sinusoidal space.

On the other hand, the distribution and percentage of CAT and Gpx1 staining were evaluated in nPQ cells of the groups studied because they had a higher expression than in PQ cells (Figure 3A–C). Although CAT and Gpx1 staining percentages are increased in nPQ cells from STZ-SME compared to STZ (*p* < 0.05), both enzymes were prominent in PQ cells of the STZ group, and there was the formation of intracellular vesicles (See Figure 3A). The percentage staining of both markers increases more in SME than in NG (*p* < 0.05). Furthermore, CAT staining appeared more prominently in the cytoplasm of PQ cells from SME than in the other groups (see Figure 3A).

**Figure 4:**
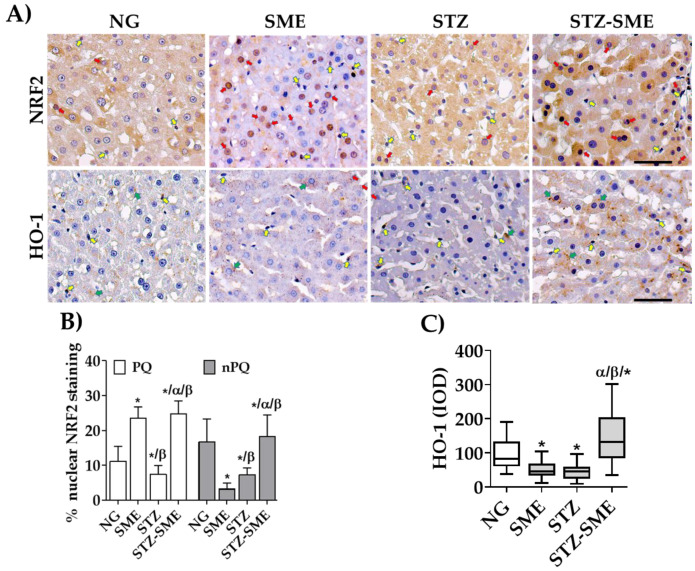
Effect of SME on the activation the cytoprotective pathway of nuclear factor E2-related factor 2/heme oxygenase 1 (NRF2/HO-1) in the livers of rats. The distribution of nuclear NRF2 and HO-1 staining in NG, SME, STZ, and STZ-SME groups is shown in panel (**A**). Panels (**B**,**C**) show the percentage of nuclear staining of NRF2 (mean ± SD) and the IOD of HO-1 (lum/pixel2) in a box-and-whisker plot (*n* = 8). The red arrows show the nuclear staining of NRF2 in PQ and nPQ cells. The yellow arrow shows nPQ cells stained positively, and the green arrow indicates cytoplasmic staining in the PQ cells. * *p* < 0.05 compared to NG; α *p* < 0.05 compared to SME; and β *p* < 0.05 compared to STZ. Scale bar = 50 μm.

The effects of SME on the hepatoprotective pathway NRF2/HO-1 were evaluated by studying its distribution in PQ and nPQ cells in the different groups (Figure 4A). In PQ cells, the percentage of nuclear staining of NRF2 was higher in the SME group compared to NG (*p* < 0.05) but not in nPQ cells, where differences were non-significant. In hyperglycemic rats, STZ-SME showed a significantly higher percentage of NRF2 nuclear staining in nPQ and PQ cells than STZ (*p* < 0.05). Additionally, the STZ-EME group displayed a higher percentage of staining in nPQ cells than in NG and SME (*p* < 0.05) (Figure 4B).

Figure 4A shows the distribution of the antioxidant enzyme HO-1 in PQ cells and nPQ cells. According to Figure 4C, the intensity of the expression of HO-1, measured in integrated optical density (IOD), is significantly higher in STZ-SME than in STZ, SME, and NG (*p* < 0.05). Additionally, the IOD values of STZ are lower than those of SME and NG (*p* < 0.05). Furthermore, there were significant differences between the NG and SME groups (*p* < 0.05).

### 3.5. Effect of SME on Inflammatory Factors in the Liver

**Figure 5:**
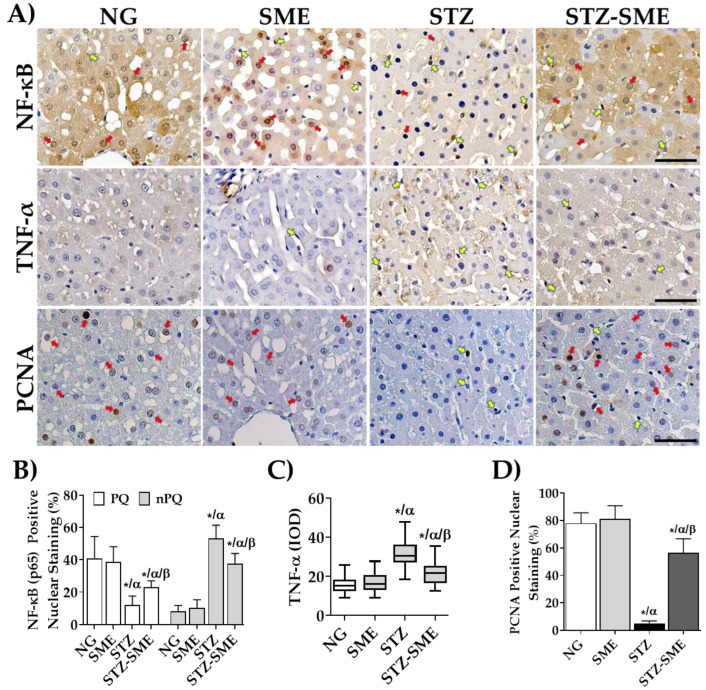
Effect of SME intake on liver inflammation. Panel (**A**) illustrates the immunostaining of NF-κB in the nuclei and tumor necrosis factor-alpha (TNF-α) in NG, SME, STZ, and STZ-SME groups. Panels (**B**–**D**) show the mean ± SD of the percentage of nuclear staining for NF-κB (PQ and nPQ cells), the IOD for TNF-α, and the percentage of nuclear staining for proliferating cell nuclear antigen (PCNA) in the PQ cells, respectively (*n* = 8). The red arrows indicate the nuclear staining in PQ cells, while the yellow arrows are in nPQ cells. * *p* < 0.05 compared to NG; α *p* < 0.05 compared to SME; and β *p* < 0.05 compared to STZ. Scale bar = 50 μm.

The activation and translocation of the NF-κB transcription factor to the nucleus in nPQ cells is caused by increased oxidative stress, leading to the expression of pro-inflammatory cytokines [37]. This study aimed to evaluate the effect of SME intake on inflammatory factors in liver tissue by evaluating NF-κB translocation to the nucleus and the expression of TNF-α. The results show a difference in the localization of NF-κB staining between PQ and nPQ cells, as illustrated in Figure 5A. The percentage of nuclear NF-κB in nPQ cells of STZ-SME is lower than in STZ (*p* < 0.05) but higher than in NG and SME (*p* < 0.05). However, no significant difference exists in the staining percentages between NG and SME.

On the other hand, the nuclear staining of NF-κB present in the PQ cells was higher in NG and SME groups as compared to STZ and STZ-SME groups (*p* < 0.05). Furthermore, the percentage of nuclear NF-κB was significantly higher in the STZ-SME group than in the STZ group (*p* < 0.05). However, there was no significant difference between the NG and SME groups. The staining intensity of TNF-α, expressed in nPQ cells, was significantly lower in STZ-SME than in STZ (*p* < 0.05) but significantly higher in STZ-SME than in NG and SME groups (*p* < 0.05), where no significant differences were observed.

Additionally, it has been observed that nuclear staining of NF-kB was found mainly in the PQ cells of the NG, SME, and SME-STZ livers (as shown in Figure 5A), which could indicate a process of cellular regeneration. Therefore, the percentage of nuclear staining of proliferating cell nuclear antigen (PCNA) in PQ cells was evaluated. Figure 5D shows a higher percentage of nuclear PCNA in NG, SME, and STZ-SME than in STZ (*p* < 0.05). However, STZ-SME was lower than NG and SME (*p* < 0.05), but there were no significant differences between NG and SME.

### 3.6. Effect of SME on Hepatic Fibrogenesis

**Figure 6:**
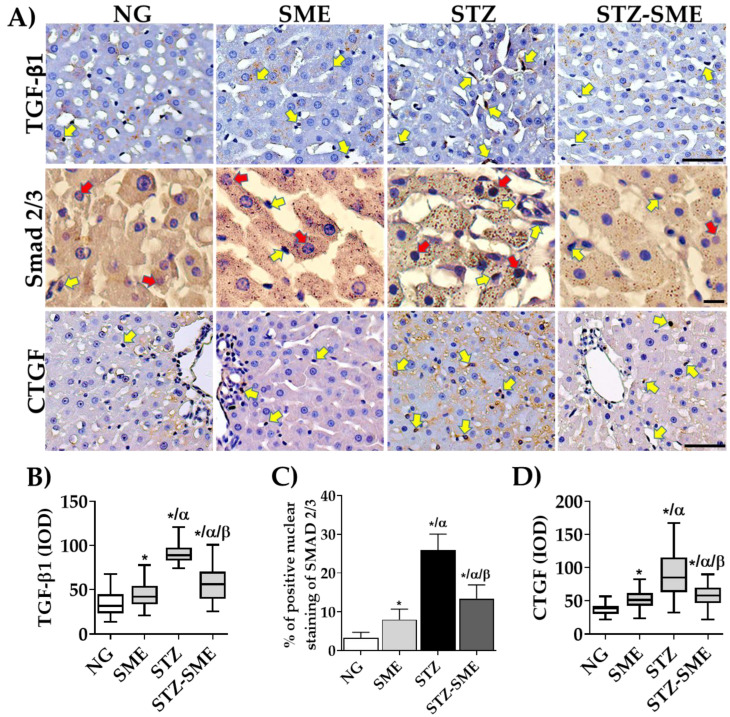
Effect of SME on liver fibrogenesis. Panel (**A**) shows representative images of immunostainings of transforming growth factor-beta1 (TGF-β1, magnifications at 400×), Smad 2/3 protein (at 800×), and connective tissue growth factor (CTGF, at 400×) in NG, SME, STZ, and STZ-SME groups. The IOD (lum/pixel2) is presented in box-and-whisker plots (panels (**B**,**D**)), and the percentage of Smad2/3 nuclear staining is shown in panel (**C**) (mean ± SD; *n* = 8). Positive staining in nPQ cells is indicated by yellow arrows and red arrows for PQ cells. * *p* < 0.05 compared to NG; α *p* < 0.05 compared to SME, and β *p* < 0.05 compared to STZ. Scale bar = 50 μm.

Excessive ROS production in nPQ, such as hepatic stellate cells, can lead to hepatic fibrogenesis by activating pro-fibrotic factors. Therefore, we determined whether SME affects the transformant growth factor-β1 (TGF-β1). As shown in Figure 6A, TGF-β1 is primarily found in the nPQ cells in the liver sinusoidal space (Figure 6A). The percentage of its staining intensity is lower in STZ-SME compared to STZ (*p* < 0.05). Additionally, In both NG and SME, the staining intensity is significantly lower than in the livers of hyperglycemic rats (*p* < 0.05) (Figure 6B).

Additionally, this study evaluated the distribution and percentage of nuclear staining of Smad2/3 (Figure 6A,C). The results revealed that in both PQ and nPQ cells, Smad 2/3 staining was present in the nucleus and the cytoplasm in all groups. However, the percentage of Smad2/3 nuclear staining was found to be higher in nPQ cells in the STZ group compared to NG, SME, and STZ-SME groups (*p* < 0.05). In the STZ-SME group, the percentage was higher than in the NG and SME groups (*p* < 0.05). The percentage of Smad 2/3 showed significant differences with an increase in SME compared to the NG group (*p* < 0.05).

The CTGF protein is a biomarker that indicates the presence of liver fibrosis. It is induced by the activation of the TGFB1/Smad2/3 pathway. We evaluated the SME effect on the CTGF expression in liver tissue by analyzing the staining intensity in nPQ cells. The results showed that the staining intensity was higher in STZ than in the STZ-SME group. However, the higher intensity of CTGF was higher in STZ-SME compared to NG and SME. Additionally, a significant increase in the SME group was observed compared to NG.

**Figure 7:**
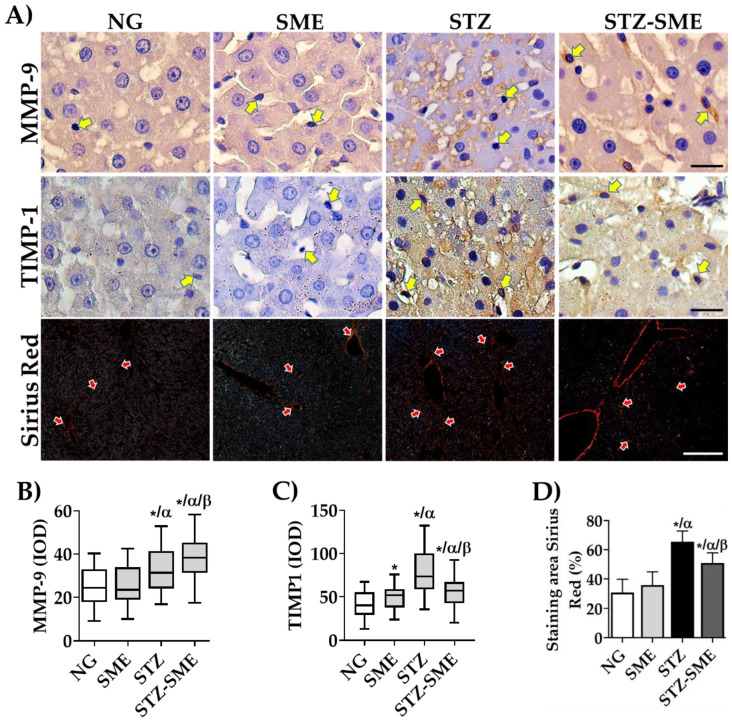
Effect of SME on the regulation of liver fibrogenesis-related factors. Panel (**A**) shows the distribution of Smad 2/3 proteins, matrix metalloproteinase-9 (MMP-9) (both scale bar = 20 μm), and collagen deposits (Sirius red stained; scale bar = 100 μm) in NG, SME, STZ, and STZ-SME. The IOD (lum/pixel2) for MMP-9 and TIMP1 are presented in box-and-whisker plots (panels (**B**,**C**); *n* = 8). The percentage of the staining area by Sirius red is shown in panel (**D**) (mean ± SD; *n* = 8). Positive staining in nPQ cells is indicated by yellow arrows, and red arrows with white borders indicate representative areas quantified. * *p* < 0.05 compared to NG; α *p* < 0.05 compared to SME, and β *p* < 0.05 compared to STZ.

This study also investigated the impact of TGF-β/SMAD3 pathway activation on liver fibrogenesis by analyzing the distribution and expression levels of tissue inhibitors of metalloproteinase-1 (TIMP1) and matrix metalloproteinase-9 (MMP-9). The findings showed that SME affected expression levels of TIMP1 and MMP-9 (Figure 7). In STZ-SME, MMP-9 staining was mainly observed in nPQ cells and the sinusoidal zone’s extracellular space (Figure 7A). In contrast, MMP-9 staining levels were significantly lower in PQ and nPQ cells in STZ (*p* < 0.05). Conversely, TIMP1 staining was higher in STZ than in STZ-SME (*p* < 0.05), with a more intense localization in nPQ cells and extracellular matrix. We observed a decrease in this marker in NG compared to the SME (*p* < 0.05). 

Liver fibrosis can be demonstrated by an increase in collagen deposits in liver tissue. Therefore, we conducted a study to determine the effect of SME treatment on collagen formation in the livers of hyperglycemic rats. We evaluated the percentage of the area of Sirius red staining to measure this effect. Our results show that the staining area in the STZ-SME group is significantly lower than in the STZ group (*p* < 0.05). However, it is still higher than in non-hyperglycemic groups. No significant differences were observed in the NG and SME groups (Figure 7A,D). This study suggests that SME has a significant effect on reducing liver fibrogenesis in hyperglycemic rats.

## 4. Discussion

Research has shown that the antioxidants in spinach can aid in preventing liver damage [20]. However, external factors such as environmental conditions during growth and cooking methods of spinach can impact its chemical composition and concentration, affecting its effectiveness [38,39]. While 100 different chemical compounds have been identified in spinach (flavonoids, flavones, flavanols, glucuronides, and carotenoids) [20,40], their quantities vary widely. To ensure consistency, we used a methanolic extract from leaves harvested from the same agricultural field containing at least ten glucopyranosides in this study [41]. To determine the antioxidant capacity of the total polyphenols in our sample, we performed assays of antioxidant activity (DPPH and ABTS), finding an IC50 that aligns with previous reports [42,43]. Our research suggests that consuming a 60 g serving of fresh spinach contains 10% polyphenols, sufficient to provide antioxidant properties with possible synergic effects [44,45]. It has been described that the beneficial effect of plant extracts on health is not based on the action of a few molecules but rather on the synergistic effect of various specific molecules that make up each plant extract [46]. This has been confirmed in the case of plant extracts that inhibit HCC growth [47,48,49].

In this study, we induced hyperglycemia in rats by administering a dose of 60 mg/kg of streptozotocin (STZ). STZ is widely used to induce an experimental model of diabetes mellitus (DM) type 1. The compound has high selectivity in its toxicity towards insulin-producing pancreatic beta cells because of its cellular uptake by low-affinity glucose transporter 2 (GLUT2). 

The underlying mechanisms of hyperglycemia contributing to liver damage come from the combination of increased oxidative stress and an aberrant inflammatory response. Other studies in animal models have shown that consuming hydroalcoholic spinach extracts counteracts liver damage caused by a high-fat diet, which is achieved by reducing serum levels of transaminases and lipid peroxidation and increasing serum levels of antioxidant enzymes [25,26,50]. In this regard, elevated levels of the enzymes AST, ALT, and ALP are a hallmark of liver damage. Currently, there have been no studies on the effects of SME on serum transaminases in animals treated with STZ. Although, there are reports on purified polyphenols that have been found in spinach, such as coumaric acid-derived and ferulic acid (3-[4-hydroxyphenyl]-2-propenoic acid) [51,52] that have antidiabetic and antilipidemic effects [53]. Polyphenols activate GLUT 2 in the pancreas, which helps to lower blood glucose levels and reduce gluconeogenic activity. This process is facilitated by increased insulin levels, which increases the activities of hexokinase and glucose-6 phosphatase dehydrogenase. Additionally, polyphenols can help decrease total cholesterol and triglyceride levels in plasma, liver, and kidney tissues [53].

Our results showed that SME treatment reduces the serum levels of AST, ALT, and ALP in rats with chronic hyperglycemia. It also lowers plasma triglyceride and cholesterol levels, suggesting that the SME diet may be beneficial in reducing these indicators and preventing liver damage. These results agreed with the report from Elvira-Torales et al. [22,50], who demonstrated that carotenoid intake from spinach and tomato had a beneficial effect by reversing steatosis in animals fed with a high-fat diet and fructose and improving the antioxidant status by reducing lipid peroxidation.

In our results, STZ-diabetic animals had increased biochemical parameters, particularly in glucose and triglyceride levels; in contrast, the cholesterol concentrations remain constant in all experimental groups, according to previous reports in diabetic animal models [54,55]. Furthermore, the administration of the SME reduced triglyceride levels by half in the animals treated with STZ, albeit they remain high when compared with NG. The glucose concentration is not significantly different between STZ and SME-STZ. Thus, these biochemical factors continue to cause liver damage, evidenced by enzyme levels.

The liver has various communication levels between cells and organs to regulate its function. However, there are currently no experimental systems capable of distinguishing the different levels of complexity, which range from the proper interaction among hepatic parenchymal (PQ) and non-parenchymal (nPQ) cells with their extracellular matrix [36]. Recent studies have shown that interactions among PQ and nPQ cells are crucial for liver regeneration and the progression of fibrogenesis [56,57,58,59]. However, it is currently unclear how spinach, its extracts, or its purified phytochemicals affect the mechanisms that protect the liver by promoting communication between PQ and nPQ cells.

Therefore, the present study investigated the relationship between PQ and non-PQ cells and their influence on the hepatic microenvironment in rats with hyperglycemia-induced liver damage, specifically on the distribution of specific oxidative, inflammatory, and fibrotic markers in STZ and how they may be regulated by consuming SME.

NADPH oxidases (NOXs) are a complex group of enzymes that produce reactive oxygen species (ROS) and whose activity can be triggered by chronic hyperglycemia [10]. Activating these enzymes may cause the release of pro-inflammatory and fibrotic factors in nPQ, while NOXs/ROS can cause cell death in PQ [12,60]. NOX4 is widely distributed in liver tissue, producing O2^•−^ and H_2_O_2_ as its primary products [61], suggesting that therapeutic targets and antioxidant agents to suppress NOXs may constitute an alternative treatment for liver fibrosis [62,63]. Hyperglycemic rats treated with SME resulted in a reduction in the formation of ROS and a decrease in the expression and distribution of NOX4 in liver cells. SME likely achieves its antioxidant activity through direct scavenging or attenuating the NOX4/ROS signaling pathways. As a result, oxidative stress-induced liver damage is reduced, as indicated by a decrease in lipid peroxidation. Therefore, SME may have a protective effect on liver cells by lowering the expression of NOX4 in nPQ cells, which may prevent liver fibrosis and cell death of PQ cells.

Recent research has shown that spinach extracts and some of their representative phytochemicals (carotenoids and lutein) provide a hepatoprotective effect by boosting the activity of the superoxide dismutase-1 (SOD1), catalase (CAT), and glutathione peroxidase (GPx1) [64,65,66]. However, when two different models—one for LPS-induced cardiac injury and another for doxorubicin-induced cytotoxicity—were treated with a water-soluble antioxidant extracted from spinach leaves (NAO), contradictory effects were found. In septic shock, NAO caused a decrease in the formation of ROS instead of triggering the activity of endogenous antioxidant enzymes. In contrast, in damage by cytotoxicity, the action of the enzymes decreased when compared to their action in the damaged group [67,68]—suggesting that the activity of NAO depends on the activation mechanism and the damage stimulus.

In the present study, we investigated the location of these antioxidant enzymes in both PQ and nPQ cells in the liver tissue of hyperglycemic rats treated with SME. Based on our observations, SOD1 is distributed throughout the liver tissue, consistent with the findings of Okado et al. [69]. The presence of SOD1 in the nucleus may be crucial for maintaining the genomic stability in nPQ cells of rats treated with SME. SOD1 acts as a transcriptional factor that regulates the expression of genes responsible for oxidative response and repair, as observed in human fibroblasts and yeast [70]. 

In hyperglycemic rats, SOD1 was present in extracellular vesicles, and in STZ-SME rats, lower levels of this enzyme were found (Figure 3). Although the role of vesicular SOD in the liver during hyperglycemia is unclear, it has been linked to neurodegenerative diseases such as amyotrophic lateral sclerosis in the nervous tissue [71]. Additionally, extracellular SOD1 causes dysfunction in the ER-Golgi compartments, leading to the death of motor neurons [72]. Our findings suggest that forming SOD1 vesicles in the liver tissue in hyperglycemic rats may contribute to hepatocyte dysfunction, likely due to a dysfunctional antioxidant capacity. Further research is needed to explore this phenomenon. 

We found that the CAT and GPx1 immunostaining was present in both PQ and nPQ cells in all groups studied, with higher levels in nPQ cells in the STZ-SME group (Figure 3). Interestingly, staining was increased in intracellular vacuoles that are larger than peroxisomes (0.1 to 1.0 μm) in the hepatocytes of hyperglycemic rats. It is currently unknown whether the CAT and GPx1 in the vacuoles have activity in the functional hepatocyte. Groothuis et al. reported that CAT inactivity is related to phospholipid–protein alterations, resulting in vesicle formation from ER membrane proliferation [73]. The process of pexophagy can cause this type of vesicle without CAT activity [74], leading to hepatocyte death in mice exposed to prolonged fasting [75]. In STZ-induced diabetes in rats, autophagy occurs in liver hepatocytes, causing the loss of ER integrity and increased degradation of endogenous proteins. The administration of insulin prevented the effects of STZ, indicating that the damage was due to insulin deficiency rather than STZ toxicity [76]. Our findings suggest that hyperglycemia-induced pexophagy injures hepatocytes and that the administration of SME can prevent this damage. More research is necessary to comprehend its mechanism. 

We also explored if the antioxidant effect of SME activates the NRF2/HO-1 cytoprotective pathway. When intracellular oxidative stress increases, NRF2 is activated and translocated to the nucleus to interact with transcription factors. It then binds to the antioxidant response element, which triggers the expression of genes responsible for phase II detoxifying enzymes, such as the antioxidant enzyme HO-1 [77].

Studies conducted in animal models have demonstrated that the NRF2/HO-1 pathway can be regulated through plant extracts or purified flavonoids to improve hepatic oxidative stress and prevent inflammation and hepatocyte apoptosis [14,78,79,80,81]. However, it remains unclear whether SME impacts the activation of NRF2/HO-1 in rats with hyperglycemia. The results of our study indicate that treatment with SME leads to a significant increase in the nuclear localization of NRF2 and expression of HO-1 in both PQ and nPQ cells in hyperglycemic rats, suggesting that the polyphenols found in the extract activate this process and offer a viable option for reducing liver damage caused by oxidative stress.

On the other hand, when nPQ cells experience an increase in oxidative stress, NRF2 is activated, and it causes the translocation of transcriptional factor NF-κB to the nucleus, leading to the expression of pro-inflammatory cytokines. A protective effect was observed in methanolic extract-treated cardiac necrosis and rats with NALFD who consumed bioactive compounds from spinach. There was a decrease in the expression of TNF-α and IL-6 in them, suggesting that the regulation occurs through the control of the NF-κB pathway [23,26,82]. These data show discrepancies with the findings of Elvira Torales et al. [22], which indicated no significant changes in inflammation and oxidative stress biomarkers, even if spinach improved the NAFLD induced by a high-fat diet in rats. Our results show that in SME-treated-hyperglycemic rats, there is a decrease in the expression of TNF-α, which is related to the percentage of the NF-κB nuclear staining in the nPQ cells (Figure 5). In addition, we also observed increases in the nuclear staining for NF-κB in the hepatocytes around the central vein, which followed the next elevation order NG > SME > STZ-SME > STZ. Therefore, this study suggests that SME significantly affects the expression of TNF-α through the inhibition of NF-κB activation in nPQ cells in the liver tissue of hyperglycemia rats. In contrast, its activation maintains the survival of PQ cells, as shown by an increase in PCNA nuclear staining [83].

When the liver is subjected to oxidative stress, HSCs are activated, leading to the expression and signaling of molecules such as TGF-β. This molecule is responsible for liver fibrosis in conditions like steatosis and cirrhosis [84]. TGF-β1 binds to TGFβRI and TGFβRII receptors, activating Smad proteins. Smad2 and Smad3 are phosphorylated and form an oligomeric complex with Smad4 that is translocated to the nucleus and transcribes genes involved in developing the extracellular matrix, such as CTGF, collagens (I, III, IV), fibronectin, and vimentin [84,85]. Our study found that treating hyperglycemic rats with SME reduced TGFβ1 staining and the percentage of nuclear staining of SMAD 2/3 in hepatocytes and nPQ cells. Besides, collagen deposits were significantly decreased, indicating that SME treatment may prevent TGF-β/SMAD signaling induced by liver fibrosis.

Furthermore, the buildup of collagen in the extracellular matrix (ECM) is related to a reduction in the activity of metalloproteinase (MMPs) and an increase in the expression of tissue inhibitors of MMPs (TIMPs) [17]. The altered inhibition by TIMP1 of the activity of MMP-9 promotes liver fibrosis [86,87,88]. We studied if the administration of SME in hyperglycemic rats affects TIMP1 and MMP-9 expression in the liver. We observed an increase in MMP-9 in the endothelium and other nPQ cells compared to that in the hyperglycemic rats. Conversely, TIMP1 was reduced, suggesting that the lost balance between MMP-9 and TIMP1 can contribute to the protective effects of SME against liver fibrosis.

## 5. Conclusions 

In STZ-induced diabetic rats, treatment with SME reduced hepatic inflammatory and fibrogenic biomarkers. Additionally, SME decreased serum transaminase levels and hypertriglyceridemia. These protective effects may be due to SME’s scavenger capacity, anti-inflammatory effect, and regulation of oxidative stress. It was observed that the effects of SME on PQ and nPQ cells were different. In nPQ cells, there was a decrease in NF-κB translocation to the nucleus, which led to a reduction in inflammatory marker expression. It also caused a decrease in the formation of free radicals and lower expression of NOX4, along with increased staining intensity of antioxidant enzymes. In PQ cells, SME activated the NRF2/HO-1 pathway, inducing a protective effect. PCNA staining in PQ cells from rats treated with SME suggests that SME has a regenerative and hepatoprotective effect. SME treatment could reduce hepatic fibrogenesis by regulating the production of the TGF-β1/SMAD 2/3 pathway and modulating the extracellular matrix. Therefore, consuming spinach could be a viable alternative to prevent severe forms of liver damage, such as NALFD and NASH, which are becoming increasingly common due to diabetes. However, more research is needed to ensure the safety of chemical compounds for human consumption.

## Figures and Tables

**Table 1:** Effect of SME on Serum Biochemical Parameters and Hepatic Enzyme Levels. The enzyme markers include glutamic-oxaloacetic transaminase (AST), glutamic-pyruvic transaminase (ALT), and alkaline phosphatase (ALP). * Indicates statistical significance with *p* < 0.05 when compared to NG; α indicates statistical significance with *p* < 0.05 when compared to SME, and β indicates statistical significance with *p* < 0.05 when compared to STZ. Values are represented in mean ± SD (*n* = 8).

Serum Levels	NG	SME	STZ	STZ-SME
ALT (U/L)	40.25 ± 7.9	49.20 ± 19.7	172.3 ± 47.4 *^/α^	105.3 ± 35.4 *^/α/β^
AST (U/L)	86.1 ± 30.8	125.3 ± 37.0	273.8 ± 57.5 *^/α^	166.8 ± 52.0 ^β^
ALP (U/L)	103.8 ± 25.4	132.5 ± 28.2	409.8 ± 149.2 *	215.8 ± 62.6 ^β^
Total cholesterol	42.7 ± 5.2	38.2 ± 5.7	51.2 ± 12.2	38.0 ± 7.4
Triglycerides (mg/dL)	39.5 ± 14.1	43.25 ± 19.2	363.6 ± 125.4 *^/α^	198.0 ± 37.9 *^/α/β^
Glucose (mmol/L)	5.44 ± 0.47	6.47 ± 1.7	26.42 ± 4.2 *^/α^	23.9 ± 1.5 *^/α^
Insulin (μUI/mL)	40.7 ± 8.1	45.3 ± 6.7 *	22.9 ± 5.1 *^/α^	25.4 ± 4.1 *^/α^

## Data Availability

The authors confirm that the data supporting the findings of this study are available within the article. Raw data are available from the corresponding author upon reasonable request.

## References

[1] King H., Aubert R.E., Herman W.H. (1998). Global burden of diabetes, 1995–2025: Prevalence, numerical estimates, and projections. Diabetes Care.

[2] Leclercq I.A., Da Silva Morais A., Schroyen B., Van Hul N., Geerts A. (2007). Insulin resistance in hepatocytes and sinusoidal liver cells: Mechanisms and consequences. J. Hepatol..

[3] Mota M., Banini B.A., Cazanave S.C., Sanyal A.J. (2016). Molecular mechanisms of lipotoxicity and glucotoxicity in nonalcoholic fatty liver disease. Metab. Clin. Exp..

[4] Gnocchi D., Sabbà C., Massimi M. (2023). Metabolism as a New Avenue for Hepatocellular Carcinoma Therapy. Int. J. Mol. Sci..

[5] Moore J.B. (2010). Non-alcoholic fatty liver disease: The hepatic consequence of obesity and the metabolic syndrome. Proc. Nutr. Soc..

[6] Xu X., Poulsen K.L., Wu L., Liu S., Miyata T., Song Q., Wei Q., Zhao C. (2022). Targeted therapeutics and novel signaling pathways in non-alcohol-associated fatty liver/steatohepatitis (NAFL/NASH). Signal Transduct. Target. Ther..

[7] Puri P., Mirshahi F., Cheung O., Natarajan R., Maher J.W., Kellum J.M., Sanyal A.J. (2008). Activation and dysregulation of the unfolded protein response in nonalcoholic fatty liver disease. Gastroenterology.

[8] Egnatchik R.A., Leamy A.K., Noguchi Y., Shiota M., Young J.D. (2014). Palmitate-induced activation of mitochondrial metabolism promotes oxidative stress and apoptosis in H4IIEC3 rat hepatocytes. Metab. Clin. Exp..

[9] Babior B.M., Lambeth J.D., Nauseef W. (2002). The neutrophil NADPH oxidase. Arch. Biochem. Biophys..

[10] Sugimoto R., Enjoji M., Kohjima M., Tsuruta S., Fukushima M., Iwao M., Sonta T., Kotoh K., Inoguchi T., Nakamuta M. (2005). High glucose stimulates hepatic stellate cells to proliferate and to produce collagen through free radical production and activation of mitogen-activated protein kinase. Liver Int. Off. J. Int. Assoc. Study Liver.

[11] Carmona-Cuenca I., Roncero C., Sancho P., Caja L., Fausto N., Fernández M., Fabregat I. (2008). Upregulation of the NADPH oxidase NOX4 by TGF-beta in hepatocytes is required for its pro-apoptotic activity. J. Hepatol..

[12] Liang S., Kisseleva T., Brenner D.A. (2016). The Role of NADPH Oxidases (NOXs) in Liver Fibrosis and the Activation of Myofibroblasts. Front. Physiol..

[13] Szypulska-Koziarska D., Wilk A., Kabat-Koperska J., Kolasa-Wołosiuk A., Wolska J., Wiszniewska B. (2019). The Effects of Short-Term Immunosuppressive Therapy on Redox Parameters in the Livers of Pregnant Wistar Rats. Int. J. Environ. Res. Public Health.

[14] Chambel S.S., Santos-Gonçalves A., Duarte T.L. (2015). The Dual Role of Nrf2 in Nonalcoholic Fatty Liver Disease: Regulation of Antioxidant Defenses and Hepatic Lipid Metabolism. BioMed Res. Int..

[15] Gharbia S., Nazarie S.R. (2022). Adipose-Derived Stem Cells (ADSCs) Supplemented with Hepatocyte Growth Factor (HGF) Attenuate Hepatic Stellate Cell Activation and Liver Fibrosis by Inhibiting the TGF-β/Smad Signaling Pathway in Chemical-Induced Liver Fibrosis Associated with Diabetes. Cells.

[16] Hu H.H., Chen D.Q., Wang Y.N., Feng Y.L., Cao G., Vaziri N.D., Zhao Y.Y. (2018). New insights into TGF-β/Smad signaling in tissue fibrosis. Chem.-Biol. Interact..

[17] Arthur M.J. (2000). Fibrogenesis II. Metalloproteinases and their inhibitors in liver fibrosis. Am. J. Physiol. -Gastrointest. Liver Physiol..

[18] Miller E.F. (2020). Nutrition Management Strategies for Nonalcoholic Fatty Liver Disease: Treatment and Prevention. Clin. Liver Dis..

[19] Pouwels S., Sakran N., Graham Y., Leal A., Pintar T., Yang W., Kassir R., Singhal R., Mahawar K., Ramnarain D. (2022). Non-alcoholic fatty liver disease (NAFLD): A review of pathophysiology, clinical management and effects of weight loss. BMC Endocr. Disord..

[20] Roberts J.L., Moreau R. (2016). Functional properties of spinach (*Spinacia oleracea* L.) phytochemicals and bioactives. Food Funct..

[21] Roman-Ramos R., Flores-Saenz J.L., Alarcon-Aguilar F.J. (1995). Anti-hyperglycemic effect of some edible plants. J. Ethnopharmacol..

[22] Elvira-Torales L.I., Periago M.J., González-Barrio R., Hidalgo N., Navarro-González I., Gómez-Gallego C., Masuero D., Soini E., Vrhovsek U., García-Alonso F.J. (2019). Spinach consumption ameliorates the gut microbiota and dislipaemia in rats with diet-induced non-alcoholic fatty liver disease (NAFLD). Food Funct..

[23] Ko S.H., Park J.H., Kim S.Y., Lee S.W., Chun S.S., Park E. (2014). Antioxidant Effects of Spinach (*Spinacia oleracea* L.) Supplementation in Hyperlipidemic Rats. Prev. Nutr. Food Sci..

[24] Bhatia A.L., Jain M. (2004). *Spinacia oleracea* L. protects against gamma radiations: A study on glutathione and lipid peroxidation in mouse liver. Phytomedicine Int. J. Phytother. Phytopharm..

[25] Amirinejad A., Hekmatdoost A., Ebrahimi A., Ranjbaran F., Shidfar F. (2020). The effects of hydroalcoholic extract of spinach on prevention and treatment of some metabolic and histologic features in a rat model of nonalcoholic fatty liver disease. J. Sci. Food Agric..

[26] Amirinejad A., Totmaj A.S., Mardali F., Hekmatdoost A., Emamat H., Safa M., Shidfar F. (2021). Administration of hydro-alcoholic extract of spinach improves oxidative stress and inflammation in high-fat diet-induced NAFLD rats. BMC Complement. Med. Ther..

[27] Li T., Lu X., Sun Y., Yang X. (2016). Effects of spinach nitrate on insulin resistance, endothelial dysfunction markers and inflammation in mice with high-fat and high-fructose consumption. Food Nutr. Res..

[28] Vutharadhi S., Jolapuram U., Kodidhela L.D. (2017). Nutraceutical inherent of Spinacia oleracea Linn. methanolic leaf extract ameliorates isoproterenol induced myocardial necrosis in male albino Wistar rats via mitigating inflammation. Biomed. Pharmacother..

[29] Bautista-Pérez R., Cano-Martínez A. (2021). Spinach Methanolic Extract Attenuates the Retinal Degeneration in Diabetic Rats. Antioxidants.

[30] Prior R.L., Wu X., Schaich K. (2005). Standardized methods for the determination of antioxidant capacity and phenolics in foods and dietary supplements. J. Agric. Food Chem..

[31] Brand-Williams W., Cuvelier M.E., Berset C. (1995). Use of a free radical method to evaluate antioxidant activity. LWT—Food Sci. Technol..

[32] Re R., Pellegrini N., Proteggente A., Pannala A., Yang M., Rice-Evans C. (1999). Antioxidant activity applying an improved ABTS radical cation decolorization assay. Free. Radic. Biol. Med..

[33] Akbarzadeh A., Norouzian D., Mehrabi M.R., Jamshidi S., Farhangi A., Verdi A.A., Mofidian S.M., Rad B.L. (2007). Induction of diabetes by Streptozotocin in rats. Indian J. Clin. Biochem. IJCB.

[34] Chun O.K., Kim D.O., Smith N., Schroeder D., Han J.T., Lee C.Y. (2005). Daily consumption of phenolics and total antioxidant capacity from fruit and vegetables in the American diet. J. Sci. Food Agric..

[35] Agoston D.V. (2017). How to Translate Time? The Temporal Aspect of Human and Rodent Biology. Front. Neurol..

[36] Godoy P., Hewitt N.J., Albrecht U., Andersen M.E., Ansari N., Bhattacharya S., Bode J.G., Bolleyn J., Borner C., Böttger J. (2013). Recent advances in 2D and 3D in vitro systems using primary hepatocytes, alternative hepatocyte sources and non-parenchymal liver cells and their use in investigating mechanisms of hepatotoxicity, cell signaling and ADME. Arch. Toxicol..

[37] Luedde T., Schwabe R.F. (2011). NF-κB in the liver—Linking injury, fibrosis and hepatocellular carcinoma. Nat. Rev. Gastroenterol. Hepatol..

[38] Palermo M., Pellegrini N., Fogliano V. (2014). The effect of cooking on the phytochemical content of vegetables. J. Sci. Food Agric..

[39] Schlering C., Zinkernagel J., Dietrich H., Frisch M., Schweiggert R. (2020). Alterations in the Chemical Composition of Spinach (*Spinacia oleracea* L.) as Provoked by Season and Moderately Limited Water Supply in Open Field Cultivation. Horticulturae.

[40] Gutierrez R.M.P., Velazquez E.G., Carrera S.P.P. (2019). Spinacia oleracea Linn Considered as One of the Most Perfect Foods: A Pharmacological and Phytochemical Review. Mini Rev. Med. Chem..

[41] Perez Gutierrez R.M., Velazquez E.G. (2020). Glucopyranoside flavonoids isolated from leaves of Spinacia oleracea (spinach) inhibit the formation of advanced glycation end products (AGEs) and aldose reductase activity (RLAR). Biomed. Pharmacother..

[42] Montenegro-Landívar M.F., Tapia-Quirós P. (2021). Recovery of Added-Value Compounds from Orange and Spinach Processing Residues: Green Extraction of Phenolic Compounds and Evaluation of Antioxidant Activity. Antioxidants.

[43] Ligor M., Trziszka T., Buszewski B. (2013). Study of Antioxidant Activity of Biologically Active Compounds Isolated from Green Vegetables by Coupled Analytical Techniques. Food Anal. Methods.

[44] El-Kersh D.M., Abou El-Ezz R.F., Fouad M. (2022). Unveiling Natural and Semisynthetic Acylated Flavonoids: Chemistry and Biological Actions in the Context of Molecular Docking. Molecules.

[45] Çelik E.E., Gökmen V. (2022). Interactions between free and bound antioxidants under different conditions in food systems. Crit. Rev. Food Sci. Nutr..

[46] Liu R.H. (2003). Health benefits of fruit and vegetables are from additive and synergistic combinations of phytochemicals. Am. J. Clin. Nutr..

[47] Gnocchi D., Del Coco L., Girelli C.R., Castellaneta F., Cesari G., Sabbà C., Fanizzi F.P., Mazzocca A. (2021). (1)H-NMR metabolomics reveals a multitarget action of Crithmum maritimum ethyl acetate extract in inhibiting hepatocellular carcinoma cell growth. Sci. Rep..

[48] Gnocchi D., Sabbà C., Mazzocca A. (2022). The Edible Plant Crithmum maritimum Shows Nutraceutical Properties by Targeting Energy Metabolism in Hepatic Cancer. Plant Foods Hum. Nutr..

[49] Thoppil R.J., Harlev E., Mandal A., Nevo E., Bishayee A. (2013). Antitumor activities of extracts from selected desert plants against HepG2 human hepatocellular carcinoma cells. Pharm. Biol..

[50] Elvira-Torales L.I., Navarro-González I., Rodrigo-García J. (2020). Consumption of Spinach and Tomato Modifies Lipid Metabolism, Reducing Hepatic Steatosis in Rats. Antioxidants.

[51] Singh A., Singh P., Kumar B., Kumar S., Dev K., Maurya R. (2019). Detection of flavonoids from Spinacia oleracea leaves using HPLC-ESI-QTOF-MS/MS and UPLC-QqQ(LIT)-MS/MS techniques. Nat. Prod. Res..

[52] Salau V.F., Erukainure O.L. (2023). Ferulic acid improves glucose homeostasis by modulation of key diabetogenic activities and restoration of pancreatic architecture in diabetic rats. Fundam. Clin. Pharmacol..

[53] Amalan V., Vijayakumar N., Indumathi D., Ramakrishnan A. (2016). Antidiabetic and antihyperlipidemic activity of p-coumaric acid in diabetic rats, role of pancreatic GLUT 2: In vivo approach. Biomed. Pharmacother..

[54] Fadzelly A.B., Asmah R., Fauziah O. (2006). Effects of Strobilanthes crispus tea aqueous extracts on glucose and lipid profile in normal and streptozotocin-induced hyperglycemic rats. Plant Foods Hum. Nutr..

[55] Lin C.H., Hsiao L.W., Kuo Y.H. (2019). Antidiabetic and Antihyperlipidemic Effects of Sulphurenic Acid, a Triterpenoid Compound from Antrodia camphorata, in Streptozotocin-Induced Diabetic Mice. Int. J. Mol. Sci..

[56] Koui Y., Kido T. (2023). Using human induced pluripotent stem cell-derived liver cells to investigate the mechanisms of liver fibrosis in vitro. Stem Cells.

[57] Zuñiga-Aguilar E., Ramírez-Fernández O. (2022). Fibrosis and hepatic regeneration mechanism. Transl. Gastroenterol. Hepatol..

[58] Fausther M., Lavoie E.G., Goree J.R., Dranoff J.A. (2017). An Elf2-like transcription factor acts as repressor of the mouse ecto-5′-nucleotidase gene expression in hepatic myofibroblasts. Purinergic Signal..

[59] Berglin L., Bergquist A., Johansson H., Glaumann H., Jorns C., Lunemann S., Wedemeyer H., Ellis E.C., Björkström N.K. (2014). In situ characterization of intrahepatic non-parenchymal cells in PSC reveals phenotypic patterns associated with disease severity. PLoS ONE.

[60] Crosas-Molist E., Fabregat I. (2015). Role of NADPH oxidases in the redox biology of liver fibrosis. Redox Biol..

[61] He W., Shi F., Zhou Z.W., Li B., Zhang K., Zhang X., Ouyang C., Zhou S.F., Zhu X. (2015). A bioinformatic and mechanistic study elicits the antifibrotic effect of ursolic acid through the attenuation of oxidative stress with the involvement of ERK, PI3K/Akt, and p38 MAPK signaling pathways in human hepatic stellate cells and rat liver. Drug Des. Dev. Ther..

[62] Mortezaee K. (2018). Nicotinamide adenine dinucleotide phosphate (NADPH) oxidase (NOX) and liver fibrosis: A review. Cell Biochem. Funct..

[63] Matuz-Mares D., Vázquez-Meza H. (2022). NOX as a Therapeutic Target in Liver Disease. Antioxidants.

[64] Panda V., Mistry K., Sudhamani S., Nandave M. (2017). Amelioration of Abnormalities Associated with the Metabolic Syndrome by Spinacia oleracea (Spinach) Consumption and Aerobic Exercise in Rats. Oxidative Med. Cell. Longev..

[65] Josson Akkara P., Sabina E.P. (2020). A biochemical approach to the anti-inflammatory, antioxidant and antiapoptotic potential of beta-carotene as a protective agent against bromobenzene-induced hepatotoxicity in female Wistar albino rats. J. Appl. Biomed..

[66] Ahn Y.J., Kim H. (2021). Lutein as a Modulator of Oxidative Stress-Mediated Inflammatory Diseases. Antioxidants.

[67] Ben-Shaul V., Lomnitski L., Nyska A., Zurovsky Y., Bergman M., Grossman S. (2001). The effect of natural antioxidants, NAO and apocynin, on oxidative stress in the rat heart following LPS challenge. Toxicol. Lett..

[68] Breitbart E., Lomnitski L., Nyska A., Malik Z., Bergman M., Sofer Y., Haseman J.K., Grossman S. (2001). Effects of water-soluble antioxidant from spinach, NAO, on doxorubicin-induced heart injury. Hum. Exp. Toxicol..

[69] Okado-Matsumoto A., Fridovich I. (2001). Subcellular distribution of superoxide dismutases (SOD) in rat liver: Cu,Zn-SOD in mitochondria. J. Biol. Chem..

[70] Tsang C.K., Liu Y., Thomas J., Zhang Y., Zheng X.F. (2014). Superoxide dismutase 1 acts as a nuclear transcription factor to regulate oxidative stress resistance. Nat. Commun..

[71] Turner B.J., Atkin J.D., Farg M.A., Zang D.W., Rembach A., Lopes E.C., Patch J.D., Hill A.F., Cheema S.S. (2005). Impaired extracellular secretion of mutant superoxide dismutase 1 associates with neurotoxicity in familial amyotrophic lateral sclerosis. J. Neurosci. Off. J. Soc. Neurosci..

[72] Sundaramoorthy V., Walker A.K., Yerbury J., Soo K.Y., Farg M.A., Hoang V., Zeineddine R., Spencer D., Atkin J.D. (2013). Extracellular wildtype and mutant SOD1 induces ER-Golgi pathology characteristic of amyotrophic lateral sclerosis in neuronal cells. Cell. Mol. Life Sci. CMLS.

[73] Groothuis G.M., Hulstaert C.E., Kalicharan D., Hardonk M.J. (1984). Genesis of unusual vesicles in rat periportal hepatocytes after administration of N-hydroxy-2-acetylaminofluorene. Virchows Archiv. B Cell Pathol. Incl. Mol. Pathol..

[74] Germain K., Kim P.K. (2020). Pexophagy: A Model for Selective Autophagy. Int. J. Mol. Sci..

[75] Dutta R.K., Maharjan Y., Lee J.N., Park C., Ho Y.S., Park R. (2021). Catalase deficiency induces reactive oxygen species mediated pexophagy and cell death in the liver during prolonged fasting. BioFactors.

[76] Lenk S.E., Bhat D., Blakeney W., Dunn W.A. (1992). Effects of streptozotocin-induced diabetes on rough endoplasmic reticulum and lysosomes of rat liver. Am. J. Physiol..

[77] Kensler T.W., Wakabayashi N., Biswal S. (2007). Cell survival responses to environmental stresses via the Keap1-Nrf2-ARE pathway. Annu. Rev. Pharmacol. Toxicol..

[78] Lee J.Y., Kim M.Y., Shin S.H., Shin M.R., Kwon O.J., Kim T.H., Park C.H., Noh J.S., Rhee M.H., Roh S.S. (2017). Persicarin isolated from Oenanthe javanica protects against diabetes-induced oxidative stress and inflammation in the liver of streptozotocin-induced type 1 diabetic mice. Exp. Ther. Med..

[79] Zhou Y., Jiang Z., Lu H., Xu Z., Tong R., Shi J., Jia G. (2019). Recent Advances of Natural Polyphenols Activators for Keap1-Nrf2 Signaling Pathway. Chem. Biodivers..

[80] Kometsi L., Govender K., Mofo Mato E.P., Hurchund R., Owira P.M.O. (2020). By reducing oxidative stress, naringenin mitigates hyperglycaemia-induced upregulation of hepatic nuclear factor erythroid 2-related factor 2 protein. J. Pharm. Pharmacol..

[81] He L., Guo C., Peng C., Li Y. (2021). Advances of natural activators for Nrf2 signaling pathway on cholestatic liver injury protection: A review. Eur. J. Pharmacol..

[82] Panda V., Bhandare N., Mistry K., Dande P. (2022). Cardioprotective potential of *Spinacia oleracea* (Spinach) against isoproterenol-induced myocardial infarction in rats. Arch. Physiol. Biochem..

[83] Michele T., Pritchard U.A. (2015). Models to Study Liver Regeneration. Liver Regeneration.

[84] Dewidar B., Meyer C., Dooley S., Meindl-Beinker A.N. (2019). TGF-β in Hepatic Stellate Cell Activation and Liver Fibrogenesis-Updated 2019. Cells.

[85] Derynck R., Zhang Y.E. (2003). Smad-dependent and Smad-independent pathways in TGF-beta family signalling. Nature.

[86] Latronico T., Mascia C., Pati I., Zuccala P., Mengoni F., Marocco R., Tieghi T., Belvisi V., Lichtner M., Vullo V. (2016). Liver Fibrosis in HCV Monoinfected and HIV/HCV Coinfected Patients: Dysregulation of Matrix Metalloproteinases (MMPs) and Their Tissue Inhibitors TIMPs and Effect of HCV Protease Inhibitors. Int. J. Mol. Sci..

[87] Hemmann S., Graf J., Roderfeld M., Roeb E. (2007). Expression of MMPs and TIMPs in liver fibrosis—A systematic review with special emphasis on anti-fibrotic strategies. J. Hepatol..

[88] Roderfeld M., Hemmann S., Roeb E. (2007). Mechanisms of fibrinolysis in chronic liver injury (with special emphasis on MMPs and TIMPs). Z. Gastroenterol..

